# Editorial: Nanotechnology and plant signaling: enhancing crop resilience to abiotic stress

**DOI:** 10.3389/fpls.2026.1974593

**Published:** 2026-09-11

**Authors:** Shalini Tiwari, Azamal Husen, Jameel M. Al-Khayri, Zienab F. R. Ahmed, Mohammad Israil Ansari

**Affiliations:** 1 Department of Natural Sciences and Mathematics, University of the Ozarks, Clarksville, AR, United States; 2 Department of Biotechnology, Graphic Era (Deemed to be University), Dehradun, Uttarakhand, India; 3 Department of Agricultural Biotechnology, College of Agriculture and Food Sciences, King Faisal University, Al-Ahsa, Saudi Arabia; 4 Integrative Agriculture Department, College of Agriculture and Veterinary Medicine, United Arab Emirates University, Al Ain, United Arab Emirates; 5 Department of Botany, University of Lucknow, Lucknow, India

**Keywords:** abiotic stress, crop resilience, nanotechnology, plant signaling, stress physiology

In the present scenario, agricultural productivity is threatened by a complex combination of abiotic stresses, including heavy-metal toxicity, herbicide injury, salinity, drought, temperature extremes, and nutrient imbalance. These stresses interrupts plant growth, photosynthesis, nutrient uptake, redox balance, and metabolism. Because climate change is increasing both the frequency and extent of these environmental factors, new techniques that increase plant resistance and maintain agricultural sustainability have to be developed. Nanotechnology has become one of the promising methods owing to the unique properties of nanomaterials that allow influencing nutrient delivery and use the antioxidant system, stress signaling, and physiology. The current Research Topic presents several papers covering different aspects, from studies on plant resistance at the nanoparticle level to plant physiology and phytohormonal signaling, bioengineered silicon nanoparticles, and nanoparticle techniques of precision genomics.

The bibliometric analysis by Luo et al. (2025) clearly demonstrates the rapid growth of research in the field of nano-agricultural sciences by analyzing 2,626 articles published from 2000 to 2023. Their research indicates that publications and citations on agricultural nanotechnology have increased exponentially over time and the focus of research has changed. While research was mainly focused on nanoparticle toxicology from 2000 to 2016, more recent studies published after 2017 deal with various aspects of agricultural nanotechnology, including nanofertilizers, nanoregulators, and nanopesticides that can optimize crop growth and enhance plants resistance to biotic and abiotic factors.

A review by Al-Dossary et al. touches upon similar developments in agriculture. They explain how nanotechnology can be helpful in increasing the resistance of crops abiotic stresses, as well as making agricultural practices more effective and sustainable. The authors describe various uses of nanomaterials, including targeted delivery of nutrients to plants and the use of nanomaterials to boost plants’ defensive mechanisms, control their physiology, and improve soil conditions, as well as to create nanosensor for plants and environmental monitoring. Moreover, the review emphases the importance of various parameters, such as particle size, structure, shape, chemical properties, and exposure time, in the success of particular nanoparticle applications. The authors also highlight potential environmental concerns associated with nanoparticle accumulation in soil and aquatic systems, including effects on microbial communities, soil enzyme activities, plant-microbe interactions, and non-target organisms. These observations emphasize that the development of nano-enabled agriculture must proceed alongside careful consideration of environmental safety, long-term behavior, and appropriate regulatory frameworks.

There are very few studies that present experimental proof regarding the ability of nanomaterials to affect plant responses to non-biological stress. Faisal et al. (2024) focused on testing the combined effect of melatonin and silicon oxide nanoparticles (SiO-NPs) on rice exposed to cadmium. The presence of cadmium resulted in oxidative stress and inhibited plant growth and function, while the use of melatonin and SiO-NPs lowered the accumulation of reactive oxygen species (ROS) and elevated antioxidant defense. An experiment performed with external application of 100 μM melatonin together with 100 mg/L SiO-NPs revealed enhanced plant growth, gas exchange, photosynthesis, and chlorophyll content. The combined treatment also significantly influenced the activity of the main antioxidant enzymes, including superoxide dismutase (SOD), catalase (CAT), and peroxidase (POX), instead of affecting only one physiological process. Therefore, the interaction of nanomaterials with endogenous or exogenous signaling molecules can improve plant tolerance to heavy-metal stress.

This topic is continued in the study by Wang et al. (2025), which was conducted to show how nanoparticles affect the reprogramming of metabolism in organisms and contribute to their tolerance to toxicity. Nicosulfuron exposure strongly impaired seedling survival, physiological characteristics, photosynthesis, and chlorophyll fluorescence, whereas foliar application of graphene oxide (GO) improved plant survival under herbicide stress. Metabolomic analysis identified 70 and 90 differentially accumulated metabolites in sweet corn inbred lines H01 and H20, respectively, following nicosulfuron exposure, while GO restored 59 metabolites in H01 and 56 metabolites in H20 toward their normal levels. These metabolites were significantly associated with survival, photosynthetic performance, and chlorophyll fluorescence, and metabolites involved in GO-mediated detoxification were enriched in flavonoid metabolic pathways. These findings highlight that the protective effects of nanoparticles do not solely depend on antioxidant enzyme activity; instead, they may induce broader metabolic adjustments that contribute to detoxification and stress adaptation. The study therefore demonstrates the value of integrating nanotechnology with metabolomics to identify biochemical pathways underlying nanoparticle-mediated stress tolerance.

Muhammad et al. (2025) studied the green-synthesized iron oxide nanoparticles (IONPs) as an alternative iron source during micropropagation of *Stevia rebaudiana*. At 5.60 mg/L, IONPs promoted early shoot and root initiation, whereas higher concentrations delayed initiation and inhibited development. However, at 22.40 mg/L, IONPs substantially enhanced leaf growth, fresh weight, and dry weight compared with the conventional FeSO_4_ treatment. IONP treatment also increased phenolic and flavonoid accumulation and antioxidant activity, with DPPH scavenging activity reaching 89.70%. These findings demonstrate that nanoparticles can function not only as nutrient-delivery systems but also as metabolic elicitors capable of modifying secondary metabolism and improving the accumulation of valuable plant products.

Another perspective on plant stress monitoring is provided by Zhang et al. (2025), who investigated the physiological and electrophysiological responses of continuously cropped *Pinellia ternata.* Continuous cropping for one to four years progressively impaired morphological development, emergence, photosynthesis, antioxidant enzyme activities and medicinal composition, while tuber yield declined by 23.84-59.20%. Importantly, the researchers also developed a comprehensive growth index and continuous-cropping obstacle index based on electrical information. These indices showed strong associations with physiological, electrophysiological, agronomic, quality, and yield parameters and provided a rapid approach for evaluating plant growth and continuous-cropping stress.

The significance of signaling mechanisms in the mediation of nanoparticles responses has been further emphasized by Ma et al., who reviewed the interactions of phytohormones and nanomaterials in plant responses to abiotic stress. Phytohormones such as abscisic acid, ethylene, gibberellins, jasmonic acid, and salicylic acid are key players in shaping plant responses to drought, salinity, extremes of temperature, and other environmental stresses. Their interactions with transcription factors and stress responsive genes allow plants to coordinate various processes related to growth, development, and stress adaptation. Of note, the review highlights hormonal communication rather than independent hormonal pathways, indicating that responses to abiotic stresses are regulated in the form of interconnected signaling networks. This framework provides an important mechanism underlying the findings demonstrated experimentally by Faisal et al. (2024), in which melatonin and SiO-NPs were used together to increase stress resistance. Future studies integrating nanoparticle treatments with comprehensive hormone profiling will be essential for determining whether nanoparticles act directly on signaling components or influence downstream physiological responses through hormonal regulation.

In an additional review, Sachdev et al. advocate for this viewpoint by analyzing bioengineered silicon nanoparticles in agro-ecosystems. They provide a detailed overview of the benefits of biologically produced SiNPs, such as minimized energy consumption, and environmentally sound, low-cost production methods. Biologically manufactured SiNPs can penetrate plants via silicic acid transporters or through endocytosis, depending on the characteristics of the particles and the types of plant. Once they enter a plant, SiNPs can improve irrigation efficiency, nutrient absorption, photosynthesis, and the plants’ antioxidant protection against dry spells, excessive heat, salinity, and heavy metals. At the molecular level, the authors showed how SiNPs may induce an appropriate ROS, followed by a stimulation of phytohormonal and MAPK signaling, leading to the expression of a series of stress-responsive genes.

Singh et al. review the development of programmable nanocarriers for precision plant engineering and describe the potential of nanoparticle systems for delivering CRISPR/Cas ribonucleoproteins, DNA, and RNA. The review covers lipid, polymeric, mesoporous silica, carbon-based, layered-double-hydroxide, and DNA-based nanocarriers, emphasizing their potential to regulate cellular uptake, cargo stability, controlled release, and tissue-specific delivery. Applications discussed include genome editing, RNA interference, organelle-targeted modification, speed breeding, meristem transformation, and multiplexed editing. This emerging field suggests that nanotechnology may ultimately move beyond protecting plants from environmental stress toward precisely modifying the genetic and regulatory networks responsible for stress resilience.

Taken together, the contributions demonstrate that nanotechnology can influence multiple interconnected layers of plant biology, including nutrient acquisition, ROS homeostasis, antioxidant defense, phytohormone signaling, photosynthesis, secondary metabolism, electrophysiological responses, and gene regulation. Al-Dossary et al. describe the ability of nanomaterials to influence ROS, antioxidant enzymes, phytohormones, and secondary metabolites while simultaneously improving nutrient acquisition and osmotic adjustment. Sachdev et al. similarly describe a signaling framework involving controlled ROS generation, phytohormones, MAPKs, and stress-responsive genes. Ma et al. place these responses within the broader context of hormonal crosstalk, while the experimental studies of Faisal et al. (2024); Wang et al. (2025), and Muhammad et al. (2025) provide concrete examples of physiological and metabolic outcomes associated with nanoparticle treatment. Overall, an important message emerging from this Research Topic is the need to move beyond single-endpoint measurements toward integrated analyses of plant signaling networks.
